# Different methods of detaching adherent cells and their effects on the cell surface expression of Fas receptor and Fas ligand

**DOI:** 10.1038/s41598-022-09605-y

**Published:** 2022-04-05

**Authors:** Ting-Yu Lai, Jerry Cao, Pu Ou-Yang, Ching-Yi Tsai, Chih-Wen Lin, Chien-Chia Chen, Meng-Kun Tsai, Chih-Yuan Lee

**Affiliations:** 1 Institute of Molecular Medicine, College of Medicine, National Taiwan University, No. 1 Jen-Ai Road, Sec. 1, Taipei, 10002 Taiwan; 2 Department of Surgery, Wollongong Hospital, Loftus Street, Wollongong, NSW 2500 Australia; 3 Department of Medical Research, National Taiwan University Hospital, No. 7 Chung-Shan South Road, Taipei, 10002 Taiwan; 4 Division of Gastroenterology and Hepatology, E-Da Dachang Hospital, and School of Medicine, College of Medicine, I-Shou University, No. 1, Sec. 1, Syuecheng Road, Dashu District, Kaohsiung City, 84001 Taiwan; 5 Department of Surgery, National Taiwan University Hospital and College of Medicine, National Taiwan University, No. 7, Chung-Shan South Road, Taipei, 10002 Taiwan; 6 Department of Surgery, National Taiwan University Hospital, Hsinchu Branch, Hsinchu City, Taiwan

**Keywords:** Biological techniques, Cell biology, Molecular biology

## Abstract

In cell culture environment, some cells adhere firmly to the culture plates and may be vulnerable to cell detachment during passage. Therefore, it is important to harvest cells with a proper detaching method to maintain the viability of cells after detachment. Trypsinization is frequently used for cellular dissociation and detachment. However, most surface proteins and the extracellular matrix are degraded by enzymatic digestion. A mild cell detachment buffer, accutase, is recommended for the replacement of trypsin to dissociate adherent cells and thereby avoid cellular damage. In this study, we demonstrated that use of accutase for cellular detachment may compromise some surface proteins. Compared with ethylenediaminetetraacetic acid (EDTA)-based nonenzymatic cell dissociation buffers, accutase was associated with significant decreases in the surface Fas ligands and Fas receptors. Moreover, we found that accutase may be able to cleave surface Fas ligands into pieces. Our results also illustrated that surface proteins required 20 h to recover after accutase treatment. We demonstrated that using accutase to dissociate adherent cells compromised the expression of Fas ligands and Fas receptors on the cell surface. These findings indicate that it is important to choose suitable cell detachment buffers and allow cells to recover after detachment before experiments.

## Introduction

Traditionally, adherent cells are cultured in monolayers adherent on flat culture plates or dishes and require detachment methods to release them before further passage or experiments. In the culture of adherent cells, cell detachment strategies are essential for cell harvest and are usually facilitated by mechanical methods combined with a calcium chelator or enzymatic cleavage of adhesion proteins to achieve effective detachment while maintaining cell viability. A variety of harvesting techniques have been employed to detach cultured adherent cells in vitro for functional and phenotypic analyses. However, the method of cell detachment may influence the surface proteins and phenotypes of cells cultured in vitro^1^. When culturing certain cells, such as macrophages, which may adhere strongly to culture dishes, certain cell detachment strategies are required to achieve effective separation while simultaneously maintaining cell survival. Cell detachment methods are also important for the stem cell maintenance of stemness^2,3^. Therefore, efficient and reliable detachment methods are critical for studying the biological characteristics of cultured adherent cells.

Some lightly adherent cells can be lifted from the dish simply by spraying with phosphate buffered saline and tapping the dish. Some strongly adherent cells require other means to break the interactions between cell proteins and the surface of the dish, thus allowing detachment from the dish. The calcium chelator ethylenediaminetetraacetic acid (EDTA) removes calcium ions that are required for integrins to maintain cell adhesion. EDTA is a mild method of cell detachment. However, it is usually not sufficiently potent for strongly adherent cells and requires mechanical dislodgement by scraping, which may inadvertently tear the cells.

Proteolytic enzyme digestion is a frequently used method for cellular detachment. However, most surface proteins and extracellular matrix components are degraded during enzymatic digestion. The loss of cell surface proteins due to excessive enzymatic degradation may influence the analysis of surface markers and reduce cell viability, especially that of stem cells^4^. The enzymes trypsin and accutase (Accutase^®^, Innovative Cell Technologies) are frequently used for detaching adherent cells. Trypsin cleaves peptides after lysine or arginine residues that are not followed by proline and degrades most cell surface proteins depending on the incubation time. Accutase is considered a mild-acting enzyme and does not affect most cell surface markers, including CD14, CD117, CD49f and CD29^2,5^. The surface levels of CD163 and CD206 were reported to be reduced in accutase-treated macrophages^1^. The Fas ligand (FasL)/Fas receptor pathway is an important mediator of cell cytotoxicity in the immune system and can also facilitate apoptosis-related cell death. We found that the surface expression of FasL was very low after the detachment of macrophages with accutase. This result confused us until we found that detaching the cells with EDTA did not decrease the surface levels of FasL.

Here, we show that the use of accutase to detach adherent cells decreases the cell surface levels of FasL and Fas receptor. This reduction in surface expression is reversible. Accutase treatment did not affect the surface expression of the murine macrophage-specific marker F4/80. Although accutase is recommended as a replacement for trypsin in the flow cytometry analysis of cell surface markers, our findings indicate that accutase cell detachment solutions should be used with caution when studying the surface expression of FasL and Fas receptor.

## Results

### Accutase decreases the surface levels of FasL and Fas receptor on macrophages

**Figure 1:**
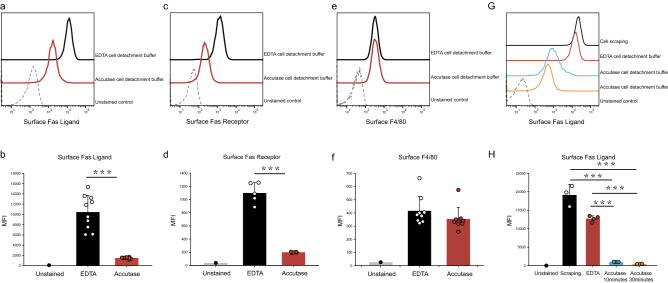
The effects of different cell detachment buffers on surface protein expression in RAW264.7 macrophages. (**a**,**c**) The surface expression of Fas ligand (FasL) and Fas receptor was detected by flow cytometry in RAW264.7 murine macrophages treated with either EDTA or accutase for 10 min. (**b**,**d**) Statistical analysis revealed a significant difference in surface FasL expression between the EDTA and accutase treatment groups. (**e**,**f**) Neither EDTA nor accutase buffer incubation affected surface F4/80 proteins in RAW264.7 cells (p = 0.2). (**G,H**) The EDTA cell detachment buffer had minor effects on cell surface FasL expression when applied for 30 min compared to those of the cell scraping method. Cells incubated with the accutase cell detachment buffer for either 10 or 30 min exhibited significantly decreased surface FasL expression. (***p < 0.001, one-way ANOVA). *MFI* mean fluorescence intensity.

We applied several cell detachment solutions, including accutase and a commercial EDTA-based nonenzymatic detachment solution (Versene, Thermo Fisher Scientific), according to the manufacturer’s instructions. The results showed that accutase dissociation solutions significantly decreased the mean fluorescence intensity (MFI) of the surface Fas ligand protein (FasL) on RAW264.7 cells in comparison to that of cells treated with the EDTA-based detachment solutions (p < 0.001) (Fig. 1a,b). We also evaluated the receptor for FasL, Fas receptor (Fas), to elucidate whether accutase would affect the surface Fas receptor amount. We found that the MFI of surface Fas was also decreased after treatment with the accutase detachment solutions (Fig. 1c,d). Interestingly, the surface level of the murine macrophage-specific marker F4/80 was not altered by accutase in comparison to that in the cells treated with the EDTA-based detachment solutions (Fig. 1e,f). In addition, we further investigated whether detachment with scraping influenced the surface expression of FasL. Our results showed that scraping tended to preserve the highest levels of surface FasL. According to the manufacturer’s instructions, cells were incubated with accutase detachment solutions for 10 min up to a maximum of 1 h, and this time was sufficient for cell detachment and passage without additional washes. Hence, we next examined whether the duration required for cellular detachment affected the surface expression of FasL. Compared to the cell scraping detachment method, macrophages incubated with EDTA-based detachment solutions for 30 min exhibited slightly decreased surface FasL expression levels (Fig. 1G,H). When macrophages were treated with accutase for 10 min, the surface levels of FasL decreased significantly compared to those in cells detached by either scraping or EDTA-based solutions. When the incubation time was increased to 30 min, the surface level of FasL did not change significantly in cells treated with EDTA-based solutions but decreased significantly in cells treated with accutase. We also treated J774A.1 cells with either the EDTA-based solution or accutase and obtained similar results (Supplementary Fig. S1). Above all, accutase detachment solutions decreased the surface levels of FasL and Fas receptor on murine macrophages in comparison to those in cells treated with the EDTA solutions. Cell detachment via scraping tended to preserve the highest surface levels of FasL.

### The effects of accutase on the surface expression of FasL and Fas receptor are reversible

**Figure 2:**
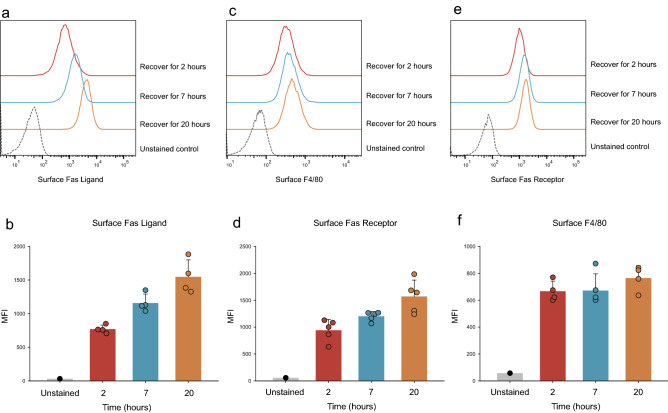
Recovery of surface proteins after treatment with accutase detachment buffer. RAW264.7 cells were incubated in accutase buffer for 30 min and then in complete medium for the indicated time amounts of time. Subsequently, the cells were harvested with EDTA detachment buffer for the analysis of surface proteins. (**a**–**d**) Significant reductions in the expression levels of the surface FasL and Fas receptor proteins were observed after recovery for 2 h in complete medium. After recovery in complete medium for 7 to 20 h, the levels of the surface FasL and Fas receptor proteins recovered gradually (p < 0.05). (**e**,**f**) No significant differences in surface F4/80 proteins were observed after treatment with accutase and recovery for the indicated time amounts of time (p = 0.33). MFI: Mean fluorescence intensity.

To determine whether the decreased surface expression of FasL proteins in accutase-treated cells could recover after removing the accutase solution, we treated macrophages with accutase for 30 min and subsequently incubated the cells in complete medium. The cells were then harvested at the indicated times for flow cytometry analysis. After recovery for 2 to 20 h, the signal intensities of surface FasL (Fig. 2a,b) and Fas receptor (Fig. 2c,d) were increased, whereas the surface levels of F4/80 were not change significantly at any time point during recovery (Fig. 2e,f). This suggested that the effects of the accutase detachment solution on the surface levels of FasL and Fas receptor were reversible, and these levels recovered after incubation for an adequate period of time.

### Accutase detachment solution helps to cleave the extracellular region of FasL

**Figure 3:**
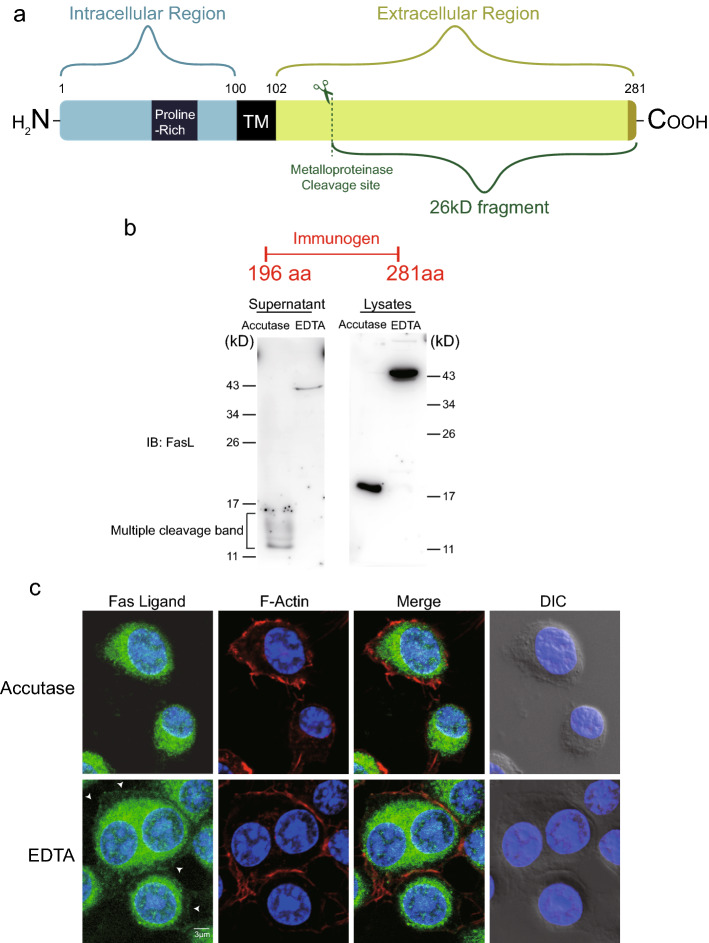
Accutase cleaved surface FasL proteins. (**a**,**b**) RAW264.7 cells were incubated with either accutase or EDTA cell detachment buffer for 30 min. The supernatants and lysates in both groups were collected and concentrated. The FasL proteins in the supernatant and lysates were analyzed by western blot. Specific antibodies against the extracellular domain of FasL were used for protein detection. (**b**) RAW264.7 cells were incubated with either accutase or EDTA cell detachment buffer for 30 min, followed by immunofluorescence staining. The white arrows indicate the FasL proteins around cell edges.

Matrix metalloproteinases (MMPs) cleave the extracellular region of FasL and release soluble FasL (Fig. 3a)^6–8^. To further investigate whether accutase inhibits the surface expression of FasL or removes its extracellular portion, we treated RAW264.7 macrophages with the accutase solution for 30 min and collected cell lysates and supernatants for western blot analysis. We used an antibody that specifically recognized the extracellular portion of FasL to detect its potential presence in the supernatant. Immunoblotting revealed several small FasL fragments under 20 kD in size in the supernatant after accutase treatment but not in the EDTA-treated cellular supernatant (Fig. 3b). We also detected a signal for full-length FasL, approximately 40 kD in size, in the EDTA-treated supernatant but not in the accutase treated supernatant. We speculate that the full-length FasL in the EDTA-treated supernatant may have originated from cells crushed during mechanical detachment with the EDTA solution. In contrast, mechanical force was usually not required when using the accutase solution to detach cells, which maintained the cell integrity and resulted in more viable cells after detachment. We also harvested lysates from the membranes of RAW264.7 cells treated with either accutase or EDTA and detected FasL by western blotting with a polyclonal antibody. The FasL in the lysate of macrophages treated with accutase was almost cleaved to fragments of 20 kD, whereas FasL in the lysate of EDTA-treated macrophages remained at approximately 40 kD (Fig. 3b). Therefore, we speculate that accutase detachment solutions help to cleave the extracellular portion of the FasL protein, which may influence the function of FasL-mediated signaling pathways. We also examined the effect of accutase detachment solutions on surface FasL by immunofluorescence staining with an antibody targeting the extracellular portion of FasL. As shown in Fig. 3c, most FasL proteins in the accutase-treated group were not localized on the cell membrane and were labeled with F-actin. In contrast, FasL remained on the cell membrane of macrophages treated with the EDTA detachment solutions, which suggested that the accutase solutions impaired the expression of FasL on the cell membranes. Collectively, these results showed that accutase solutions impair the surface expression of FasL on macrophages and remove the extracellular portion of FasL.

**Figure 4:**
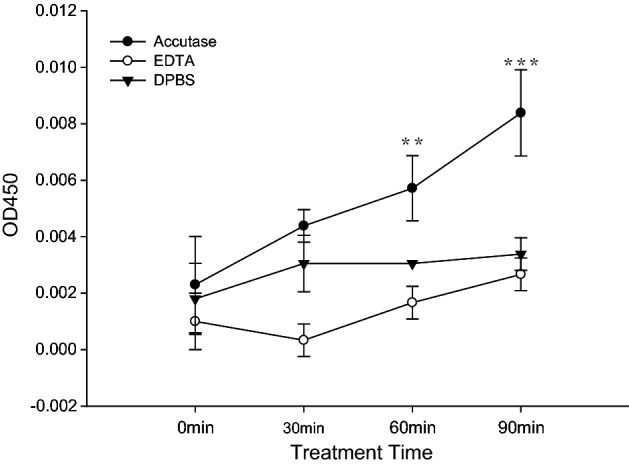
The proliferation of cells treated with different detachment buffers was detected by the CCK-8 assay. RAW264.7 macrophage proliferation was significantly increased by treatment with accutase for 30, 60 (p < 0.01) and 90 min (p < 0.001); *compared with EDTA detachment buffer and DPBS.

We further investigated whether cell detachment with accutase would promote cell viability. We used the CCK-8 assay to determine the viability of cells after cell detachment with different solutions. The viable cell counts were increased in the group treated with the accutase solution for 60 min compared to those in the groups treated with the EDTA solution or DPBS buffer (p < 0.01) (Fig. 4). Even after 90 min of treatment, the cells in the accutase-treated group were significantly more viable than those in the other groups (p < 0.001). This suggested that the accutase detachment solutions maintain cell viability after cell detachment better than the other solutions. We could not confirm the relationships among cell viability after detachment, cleaved surface FasL, and Fas receptor.

## Discussion

Accutase is commonly used in the detachment of adherent cells for flow cytometry, as it is widely believed to be gentler and less problematic for the detection of surface markers. Before knowing the effects of accutase on several specific cell surface proteins, including FasL and Fas receptor, we obtained some confusing experimental results when studying the surface expression of FasL on macrophages. We examined F4/80 on the surface of macrophages by flow cytometry and found that the expression was not altered by accutase detachment, which made us feel comfortable using accutase as an alternative to trypsin as a mild-acting cell detachment solution. After comparing the surface expression levels of FasL on macrophages detached by either accutase or EDTA solutions, we observed that using accutase decreased the surface expression of FasL. We also found that the surface expression of Fas receptor was decreased on macrophages, but that of F4/80 was not changed after treatment with accutase. According to published reports, accutase decreases the expression of only a limited number of cell surface proteins, including CD163 and CD206^1^. Here, we report another 2 surface proteins, FasL and Fas, whose expression was decreased after treatment with accutase. Several proteins, such as CD14, are affected by trypsin but not by accutase. The surface expression of CD14, CD200R, CD80, CD86, CD16, CD32, and CD64 is not affected by trypsin, EDTA, or accutase^1^. Therefore, it is important to choose an appropriate detachment method according to the study targets and experimental designs.

According to our results, the effect of accutase on FasL was reversible, and the expression of FasL on the cell surface recovered 24 h after treatment. Therefore, it is necessary to wait for the recovery of surface proteins in cells treated with accutase before using them in experiments. FasL on the cell membrane can reportedly be cleaved by MMPs and release a 26 kD soluble peptide called soluble FasL (sFasL)^6^, which binds the Fas death receptor^9^ and modulates immune responses^10–12^. MMP inhibitors can block the release of sFasL, leading to the accumulation of FasL on the cell surface and to the apoptotic induction of the surrounding Fas-sensitive cells^13^. We herein found that FasL in the lysates of cells treated with accutase was smaller than that in the lysates of cells treated with EDTA detachment solutions when using a FasL polyclonal antibody. When we used an antibody that could recognize the extracellular portion of FasL, we detected multiple small fragments in the supernatant of cells treated with accutase but not in that of cells treated with the EDTA detachment solutions. All MMPs are synthesized and secreted as proenzymes that are activated by a variety of proteases^14^. From our results, we could not draw a conclusion on whether the surface FasL was cut directly by accutase or indirectly by the activation of MMPs. Therefore, we speculate that accutase treatment helps to remove the extracellular portion of FasL from the cell membrane.

According to published reports, membrane-bound FasL is more active than soluble FasL in inducing the apoptosis of T cells^15,16^. Apoptosis is important for regulating the immune response. The binding of FasL to Fas locally via cell–cell interactions and the formation of Fas microaggregates at the cell surface could induce apoptosis in Fas-bearing cells, which is the fundamental principle of the Fas-FasL-mediated apoptosis assay^17^. Therefore, the use of accutase in the Fas–FasL mediated apoptosis assay may interfere with the results. Defective Fas or FasL functions lead to autoimmune lymphoproliferative syndrome (ALPS), which is a nonmalignant and noninfectious uncontrolled proliferation of lymphocytes^18^. Demonstrating the defect of Fas–FasL mediated apoptosis in the lymphocyte apoptotic assay is critical for the diagnosis and study of ALPS, which may also be affected by the use of accutase in the assay. Cancer cells may use FasL expression as a means to escape immune surveillance. In this situation, tumor cells induce the FasL-mediated apoptosis of Fas-expressing tumor-infiltrating lymphocytes. This hypothesis was supported by the finding that colon cancer cells expressing FasL induced the apoptosis of T cells in an in vitro apoptotic assay^19^, which may have also been influenced by accutase.

The loss of cell surface proteins due to enzymatic degradation by the detachment solution reportedly affected the survival of stem cells and cultured epithelial cells after passage^5^. Inhibition of metalloproteinases may enhance the cytotoxic activity of FasL^6^. During cell passage, usage of the cell detachment solution accutase was also reported to inhibit the apoptosis of human embryonic stem cells^20,21^. Our data also demonstrated that more viable cells were obtained when accutase rather than other methods was used to detach adherent macrophages, which have might been due to the removal of FasL from the cell surface after treatment with accutase. However, further experiments are necessary to confirm this result.

## Conclusion

In summary, we found that detaching cultured macrophages with accutase decreases the surface expression of FasL and Fas receptor. The expression of FasL on the cell membrane recovered 24 h after incubation.

## Methods

### Cell culture

The mouse macrophage-like cell line RAW264.7 was obtained from ATCC (Manassas, VA) and cultured at 37 °C and 5% CO2 in RPMI (Gibco BRL, Grand Island, NY) supplemented with 10% fetal bovine serum (FBS, US origin, Gibco). RAW264.7 cells were maintained via weekly passage and utilized for the experiment at 60–80% confluence.

### Cell detachment method

RAW264.7 cells (1 × 10^6^ cells per 6 cm dish) were incubated overnight at 37 °C and 5% CO2 in a 6 cm dish to facilitate attachment and spreading before experiments. J774A.1 cells were cultured in Dulbecco’s modified Eagle’s medium (DMEM) supplemented with 10% fetal bovine serum. Before experiments, the cells were washed with DPBS. Adherent macrophages were then detached via either accutase (Innovative Cell Technologies, San Diego, CA), a commercially available EDTA detachment solution (Versene, Gibco, Grand Island, NY), or scraping. After 10–30 min, the cells were stained with immunofluorescence or harvested for western blot and fluorescence-activated cell sorting (FACS) analyses. The cell supernatant was collected and concentrated with an Amicon^®^ Ultra15 centrifugal filter for evaluation by western blotting.

### Cell lysate and supernatant protein lysis

The cells were rinsed twice with ice-cold PBS and covered with a cell lysis buffer containing 50 mM Tris/HCl pH 7.4, 1% Triton X-100, 1% sodium deoxycholate with complete™ Mini EDTA-free Protease Inhibitor Cocktail and PhosSTOP Phosphatase Inhibitor Cocktail (Roche Diagnostics GmbH, Mannheim, Germany). Concentrated proteins from the cell supernatant were also lysed with lysis buffer. After scraping, the cells were sonicated for 10 min and centrifuged at 15,000×*g* for 15 min at 4 °C. Both samples were boiled for 5 min at 70 °C in 4× NuPAGE LDS Sample Buffer (Novex, Invitrogen) with 5% β-mercaptoethanol and analyzed by western blot. An anti-Fas ligand polyclonal antibody (bs-0216R, Bioss Antibodies, Woburn, MA) was used for protein detection in cell lysates and supernatant.

### Flow cytometry

The detection of accessory molecule expression on RAW264.7 cells by FACS analysis was performed as follows. Briefly, RAW264.7 cells were harvested by accutase or EDTA dissociation buffer and washed with FACS buffer (3% bovine serum albumin (BSA) and 0.1% sodium azide in DPBS). The cells were stained with the optimal concentrations of fluorochrome-conjugated antibodies for 30 min. The antibodies used in these experiments were Fas ligand-APC (eBioscience, San Diego, CA), CD95-FITC (BD Pharmingen, San Diego, CA), and F4/80-FITC (clone: BM8; BioLegend, San Diego, CA). One hundred thousand cells per treatment were acquired and analyzed using a BD FACSVerse flow cytometer and FlowJo software (version 10.7, BD Ashland, OR). Cells were gated using forward scatter vs. sideward scatter properties to exclude debris and doublets. The mean fluorescence intensity (MFI) of the whole macrophage population was analyzed.

### Immunofluorescence

Cells were fixed with methanol for 10 min, blocked with 1% BSA for 2 h, and then incubated with Fas ligand primary antibodies (Bioss Antibodies, Woburn, MA) for 16 h at 4 °C. After 3 washes with PBS, the cells were incubated with secondary antibodies and rhodamine phalloidin in 1% BSA for 2 h. The nuclei were stained with Hoechst 33342. After 3 washes with PBS, the cells were mounted with Fluoromount G (Southern Biotech, Birmingham, AL) antifade reagent and imaged with a confocal microscope (SP8X, LEICA).

### Cell counting kit-8 assay

Cell proliferation was detected using the Cell Counting Kit-8 (CCK8) assay (Dojindo Molecular Technologies, Kumamoto, Japan) following the manufacturer’s protocol. In brief, cells were plated in 24-well plates (10^5^ cells/1 mL/well) and treated with different buffers for the indicated amounts of time, with three duplicate wells for each subgroup. The incubation buffer without cells served as the blank control. The plates were incubated at 37 °C and 5% CO2. A total of 100 μL of CCK-8 reagent was added to the corresponding wells at each time point and incubated for 1 h. Later, the optical density (OD) was measured using a microplate reader (Molecular Devices, San Jose, CA) at a wavelength of 450 nm.

### Statistics

The data are presented as the mean ± SD, and the differences between treatment groups were determined by one-way analysis of variance (ANOVA).

## Supplementary Information

Supplementary Figures.

## Data Availability

The data that support the findings of this study are available from the corresponding author upon reasonable request.
