# Supplementary material for: Different methods of detaching adherent cells and their effects on the cell surface expression of Fas receptor and Fas ligand
Source: Sci Rep. 2022 Apr 5;12:5713. doi: 10.1038/s41598-022-09605-y (PMC8983651; doi:10.1038/s41598-022-09605-y)

Supplementary Figure 1

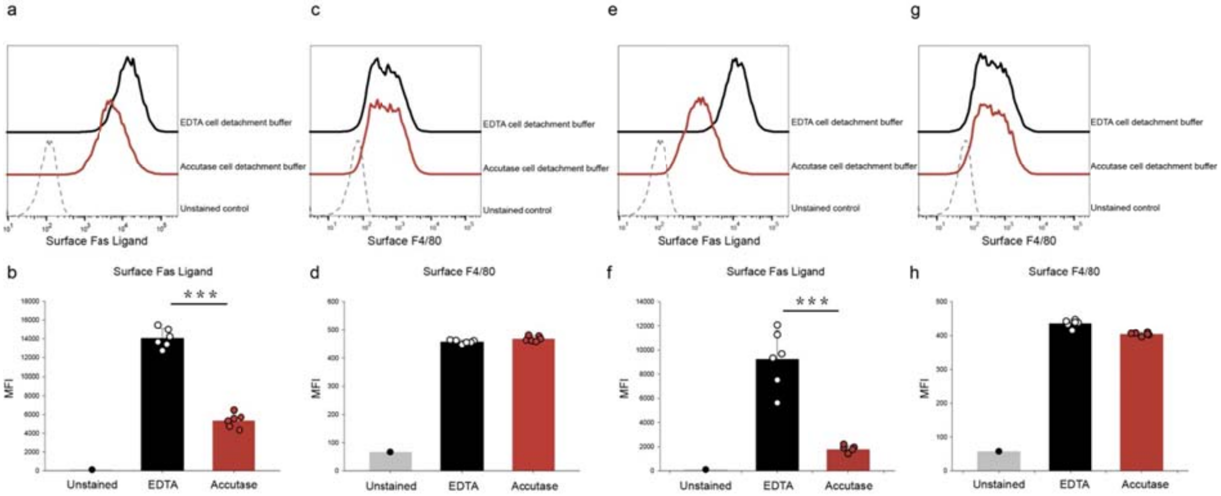

Supplementary Figure 1
The effects of different cell detachment buffers on surface protein expressions in J774A.1 macrophages. (a, c) The surface expressions of Fas ligand (FasL) and F4/80 were detected by flowcytometry on J774A.1 murine macrophages treated with either EDTA or accutase for 10 minutes. (b, d) Quantified resultsfrom (a) and (c) showed significant differences in surface FasL expression on J774A.1 cells between treatment with EDTA detachment buffers or accutase ($p < 0.001$), but no differences were detected in surface F4/80 expression. (e, g) We increased the time of treatment with detachment buffers to 30 minutes. The surface expressions of FasL and F4/80 were detected by flowcytometry on J774A.1 murine macrophages treated with either EDTA detachment buffers or accutase. (f) The surface expression of FasL was still lower on J774A.1 cells treated with accutase than those treated with EDTA detachment buffers.(g)The differences in surface F4/80 expression on J774A.1 cells treated with either EDTA detachment buffers or accutase were not significantly different (***: $p < 0.001$).

Supplementary file for Figure 3b

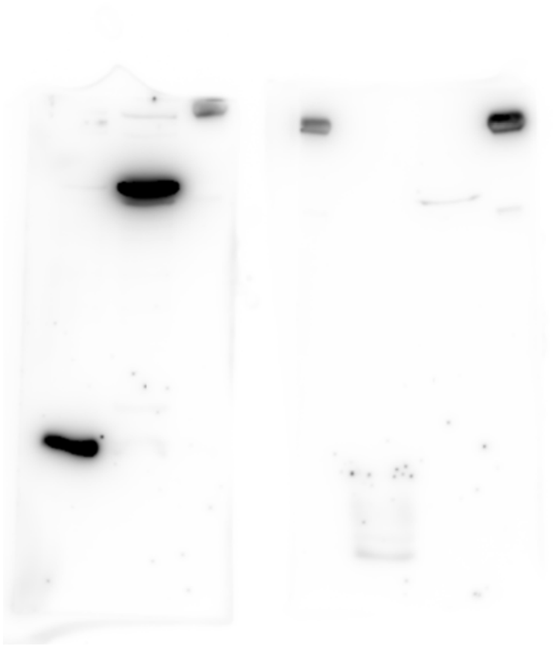

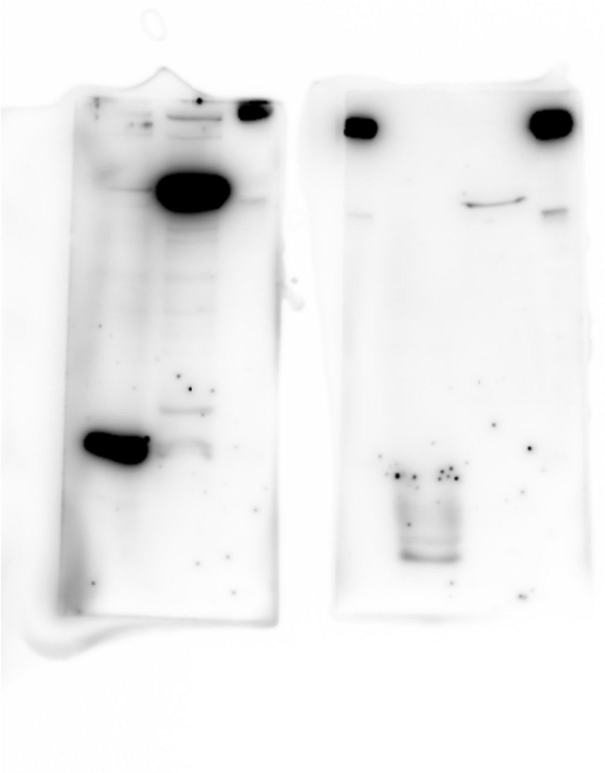

Supplement: Supplementary file 1 — Supplementary Figures. [file 41598_2022_9605_MOESM1_ESM.pdf]
